# Vascular Endothelial Growth Factor Improves Physico-Mechanical Properties and Enhances Endothelialization of Poly(3-hydroxybutyrate-co-3-hydroxyvalerate)/Poly(ε-caprolactone) Small-Diameter Vascular Grafts *In vivo*

**DOI:** 10.3389/fphar.2016.00230

**Published:** 2016-07-29

**Authors:** Larisa V. Antonova, Victoria V. Sevostyanova, Anton G. Kutikhin, Andrey V. Mironov, Evgeniya O. Krivkina, Amin R. Shabaev, Vera G. Matveeva, Elena A. Velikanova, Evgeniya A. Sergeeva, Andrey Y. Burago, Georgiy Y. Vasyukov, Tatiana V. Glushkova, Yuliya A. Kudryavtseva, Olga L. Barbarash, Leonid S. Barbarash

**Affiliations:** Research Institute for Complex Issues of Cardiovascular DiseasesKemerovo, Russia

**Keywords:** poly(3-hydroxybutyrate-co-3-hydroxyvalerate), poly(ε-caprolactone), vascular endothelial growth factor, vascular graft, morphology, physico-mechanical properties, endothelialization, patency

## Abstract

The combination of a natural polymer poly(3-hydroxybutyrate-co-3-hydroxyvalerate) (PHBV) and a synthetic hydrophobic polymer poly(ε-caprolactone) (PCL) is promising for the preparation of biodegradable and biocompatible small-diameter vascular grafts for bypass surgery. However, physico-mechanical properties and endothelialization rate of PHBV/PCL grafts are poor. We suggested that incorporation of vascular endothelial growth factor (VEGF) into PHBV/PCL grafts may improve their physico-mechanical properties and enhance endothelialization. Here we compared morphology, physico-mechanical properties, and *in vivo* performance of electrospun small-diameter vascular grafts prepared from PHBV/PCL with and without VEGF. Structure of the graft surface and physico-mechanical properties were examined by scanning electron microscopy and universal testing machine, respectively. Grafts were implanted into rat abdominal aorta for 1, 3, and 6 months with the further histological, immunohistochemical, and immunofluorescence examination. PHBV/PCL grafts with and without VEGF were highly porous and consisted mostly of nanoscale and microscale fibers, respectively. Mean pore diameter and mean pore area were significantly lower in PHBV/PCL/VEGF compared to PHBV/PCL grafts (1.47 μm and 10.05 μm^2^; 2.63 μm and 47.13 μm^2^, respectively). Durability, elasticity, and stiffness of PHBV/PCL grafts with VEGF were more similar to internal mammary artery compared to those without, particularly 6 months postimplantation. Both qualitative examination and quantitative image analysis showed that three-fourths of PHBV/PCL grafts with VEGF were patent and had many CD31-, CD34-, and vWF-positive cells at their inner surface. However, all PHBV/PCL grafts without VEGF were occluded and had no or a few CD31-positive cells at the inner surface. Therefore, VEGF enhanced endothelialization and improved graft patency at all the time points in a rat abdominal aorta replacement model. In conclusion, PHBV/PCL grafts with VEGF have better biocompatibility and physico-mechanical properties compared to those without. Incorporation of VEGF improves graft patency and accelerates formation of endothelial cell monolayer.

## Introduction

Reconstructive surgery is a conventional treatment of coronary artery disease and peripheral artery disease, and autologous saphenous vein (SV), internal mammary and radial artery grafts are commonly used (Taggart, 2013). However, a significant proportion of the patients do not have suitable veins or arteries that could be used (Desai et al., 2011). Therefore, tissue engineering of vascular grafts is a promising approach for the replacement of small-diameter (<6 mm) blood vessels (Desai et al., 2011). Small-diameter vascular grafts can be prepared from biodegradable polymers and may become a scaffold for the formation of a blood vessel *in situ* (Antonova et al., 2015a). The combination of natural and synthetic polymers was suggested as improving the properties and resorption rate of the scaffold (Antonova et al., 2015a).

It was demonstrated that poly(ε-caprolactone) (PCL) may be successfully used for the preparation of the vascular grafts (de Valence et al., 2012; Kuwabara et al., 2012). Electrospun PCL grafts implanted into rat abdominal aorta possessed structural integrity, patency, and promoted formation of extracellular matrix 6 months postimplantation (Pektok et al., 2008). Poly(3-hydroxybutyrate-co-3-hydroxyvalerate) (PHBV) is a natural polymer synthesized by bacteria as storage compound under growth limiting conditions (Quillaguamán et al., 2010). One of its monomers, 3-hydroxybutanoic acid, is a natural metabolite produced in the human body (Quillaguamán et al., 2010). This ensures a high biocompatibility of PHBV (Quillaguamán et al., 2010). According to the previously published data, PHBV/PCL combination improves biocompatibility of electrospun small-diameter vascular grafts (Del Gaudio et al., 2012; Antonova et al., 2015b).

Vascular endothelial growth factor (VEGF) is a key player in vasculogenesis and angiogenesis, and therefore it is widely used for the stimulation of graft endothelialization (Maes et al., 2002). VEGF induces migration, proliferation, and survival of endothelial cells, enhances nitric oxide production, and improves vascular permeability (Takahashi et al., 2004). We previously demonstrated that VEGF incorporated into electrospun polymer scaffolds retains its biological activity *in vitro* (Sevostyanova et al., 2015). Here we compared morphology, physico-mechanical properties, patency and endothelialization rate of PHBV/PCL small-diameter vascular grafts with and without VEGF after the implantation into rat abdominal aorta.

## Materials and Methods

### Graft Preparation

Small-diameter vascular grafts were prepared by electrospinning (Nanon-01A, MECC) from PHBV/PCL (1:2, Sigma–Aldrich)/chloroform solution using the following parameters: 20 kV voltage, 0.3 mL/h feed rate, 2 mm rotating drum diameter, and 22G needle. VEGF (Sigma–Aldrich) was dissolved in phosphate buffered saline (PBS) to 10 μg/mL concentration and then added to PHBV/PCL/chloroform solution (1:20) with the final concentration of 500 ng/mL. For the preparation of electrospun PHBV/PCL/VEGF grafts, we used the following parameters: 23 kV voltage, 0.3 mL/h feed rate, 2 mm rotating drum diameter, and 27G needle.

### Morphological Assessment

PHBV/PCL and PHBV/PCL/VEGF graft samples 0.5 mm × 0.5 mm (*n* = 5 per group) were examined using scanning electron microscopy (Hitachi S-3400N, Hitachi) with Au-Pd sputter coating (Quorum Technologies) of 30 nm thickness. Fiber diameter, pore area, and porosity were measured using ImageJ (National Institutes of Health). Mean fiber diameter and mean pore area were calculated after at least 100 measurements per each sample.

### Evaluation of Physico-Mechanical Properties

Assessment of durability and elastic deformation properties was performed using universal testing machine (Zwick/Roell). Testing was performed with 1 cm working segment length, 0.01 N preload, and 10 mm/min crosshead speed. Durability, elasticity, and stiffness were evaluated by yield stress, relative elongation, and elastic modulus, respectively. We assessed six vascular grafts per group. Synthetic vascular graft made of expanded poly(tetrafluoroethylene) (ePTFE, Vascutek), human SV and internal mammary artery (IMA) (*n* = 6 per each) were used as the controls. SV and IMA were collected from patients who underwent coronary artery bypass graft surgery, and all the participants provided written informed consent after receiving a full explanation of the study. The study protocol was approved by a local ethical committee.

### *In vivo* Implantation

All animal experiments were performed in 6-month-old male Wistar rats (400–450 g body weight, *n* = 24) according to all official and ethical requirements. Rats were obtained from the core facility of Research Institute for Complex Issues of Cardiovascular Diseases. The animals were allocated in polypropylene cages (five animals per cage) lined with wood chips and had access to water and food (rat chow) *ad libitum.* Throughout the whole time of experiment, standard conditions of temperature (24 ± 1°C), relative humidity (55 ± 10%), and 12 h light/dark cycles were carefully maintained, and the health status of all rats was monitored daily.

After ethylene oxide sterilization, 2 mm diameter and 10 mm length PHBV/PCL and PHBV/PCL/VEGF (*n* = 12 per group) grafts were implanted into rat abdominal aorta after the induction of anesthesia with 3% isoflurane. During the surgery, all animals received inhalation anesthesia with 1.5% isoflurane. Briefly, a midline laparotomy was performed. After the isolation of the abdominal cavity with a sterile cloth, intestinal loops were moved to the right and wrapped in a wet warm cloth. The posterior peritoneal leaflet was opened along the mesenteric root, and the aorta was mobilized from the level of renal arteries to bifurcation. Then, aorta was temporarily occluded with two microvascular bulldog clamps distally from the renal arteries and proximally from the bifurcation. The aorta and vena cava inferior were occluded in parallel. Both proximal and distal anastomoses were performed using Prolene 8-0. Graft was washed between the performances of these anastomoses. After the implantation, the anterior abdominal wall was closed layer-by-layer with a blanket suture (4-0 or 2-0 Vicryl). All procedures were performed using strict aseptic technique. In each group, one-third (*n* = 4) of rats was sacrificed 1, 3, and 6 months postimplantation.

After the explantation, grafts with the adjacent aortic tissue were divided into two equal parts (5 mm length each). The first part was frozen at -140°C for the further immunofluorescence staining. The second part was fixed in 10% (w/v) neutral phosphate buffered formalin (Electron Microscopy Sciences) during 24 h at 4°C for the further histological and immunohistochemical examination.

### Histological Examination

After the explantation, formalin-fixed grafts were dehydrated in isopropanol during 30 h at 4°C, rinsed in distilled water, embedded in paraffin (Electron Microscopy Sciences), sectioned (5 μm), and finally mounted on glass microscope slides. For a deparaffinization, paraffin-embedded tissue sections were heated in dry oven at 60°C for 20 min, immersed in the following reagents: 3x xylene (Electron Microscopy Sciences) for 10 min, 100, 95, 70, 50, 30% ethanol for 1 min each, physiological saline for 2 min, PBS for 2 min, and finally rinsed with tap water. For hematoxylin and eosin (H&E) staining, the sections were immersed in Harris Hematoxylin solution (Electron Microscopy Sciences) for 1 min, rinsed with tap water, immersed in 1% aqueous Eosin Y solution (Electron Microscopy Sciences) for 1 min, rinsed with tap water, dehydrated in ascending ethanol solutions (50, 70, 80, 2x 95%, and 2x 100%), and then cleared 2x with xylene. Coverslips were mounted onto a labeled glass slide with Permount (Electron Microscopy Sciences). After the staining, sections were evaluated by light microscopy (Axio Imager A1, Carl Zeiss) in a blinded fashion; three sections per stain were assessed from each rat.

### Immunohistochemistry

For immunohistochemical assessment, we used the kit of Novocastra (Thermo Scientific) and rabbit anti-CD31 antibodies (M338, Spring Bioscience) according to manufacturer’s instructions. Briefly, samples were boiled in citrate buffer (0.01 M, pH 6.0) for antigen retrieval, treated by the inhibitor of endogenous peroxidase for 10 min, and washed twice in phosphate buffer (0.01 M, pH 7.4) for 5 min. For blocking non-specific background staining, we treated the samples with Protein Block for 10 min with the further 2x washing in phosphate buffer for 5 min. Then, we stained the samples with 50 μL of primary anti-CD31 (PECAM-1) antibodies, incubated them in a wet chamber for 1 h, and performed the staining with goat anti-rabbit secondary antibodies (12-348, Millipore). After the washing in phosphate buffer, we treated samples with streptavidin-peroxidase conjugate and diaminobenzidine with the further assessment of the reaction which was stopped by cold distilled water. Slides were then stained with Mayer’s Hematoxylin and finally mounted. Native blood vessels and antibody diluent were used as a positive and negative control, respectively. After the staining, sections were evaluated by light microscopy (Axio Imager A1, Carl Zeiss) in a blinded fashion; three sections per stain were assessed from each rat.

### Confocal Laser Scanning Microscopy

Snap-frozen tissue blocks were cut on a cryostat (Microm HM 525, Thermo Scientific), and sections (8 μm) were mounted on the glass microscope slides. Samples were stained with unconjugated mouse anti-CD31 (ab119339, Abcam) and rabbit anti-CD34 (ab185732, Abcam) or fluorescein isothiocyanate (FITC)-conjugated sheep anti-von Willebrand factor (vWF, ab8822, Abcam) primary antibodies and incubated at 4°C for 17 h. Then, in the case of CD31 and CD34 staining, slides were treated with secondary sheep anti-mouse rhodamine-conjugated (AP300R, Millipore) and goat anti-rabbit FITC-conjugated antibodies (12-507, Millipore) with the further incubation for 1 h at room temperature. Washing was performed thrice with PBS. Autofluorescence Eliminator Reagent (Millipore) was used to reduce autofluorescence. If needed, nuclei were stained with DAPI for 30 min at room temperature (10 μg/mL, Sigma–Aldrich). Slides were mounted onto a labeled glass slide with ProLong (Life Technologies). Native rat aorta was used as a positive control for anti-CD31 and anti-vWF staining whereas rat embryo was used as a positive control for anti-CD34 staining. Bovine serum albumin (Sigma) was used instead of the primary antibodies as a negative control. Visualization was performed using LSM 700 Confocal Laser Scanning Microscope (Carl Zeiss) in a blinded fashion; three sections per stain were assessed from each rat. Quantitative image analysis was performed using ImageJ.

### Statistical Analysis

Statistical analysis was performed using GraphPad Prism (GraphPad Software). A sampling distribution was assessed by D’Agostino-Pearson test and Kolmogorov–Smirnov test. Depending on the distribution, descriptive data were represented by median and interquartile range (25th and 75th percentiles) or mean and standard deviation of the mean. Two independent groups were compared by Mann–Whitney *U*-test or two-tailed Student’s *t*-test. Independent groups numbering three or more were compared using Kruskal–Wallis test or analysis of variance (ANOVA), with pairs further compared by Mann–Whitney *U*-test or two-tailed Student’s *t*-test if statistically significant differences were revealed by Kruskal–Wallis test or ANOVA, respectively. An adjustment for multiple comparisons was performed using false discovery rate (FDR). *P*-values, or *q*-values if FDR was applied (*q*-values are the name given to the adjusted *p*-values found using an optimized FDR approach), ≤0.05 were regarded as statistically significant.

## Results

### Incorporation of VEGF into PHBV/PCL Vascular Grafts Leads to the Formation of Nanoscale Fibers and Small Pores

**FIGURE 1:**
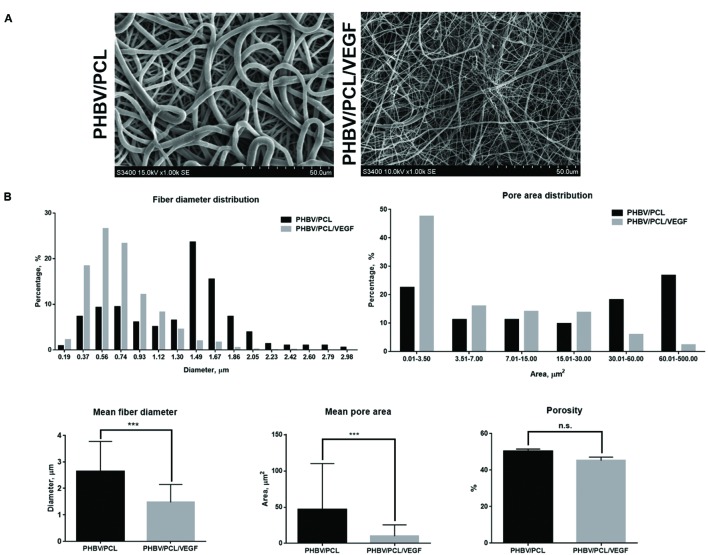
**Morphology of PHBV/PCL vascular grafts with and without VEGF. (A)** Scanning electron microscopy images; **(B)** Quantitative image analysis (five images containing in total >100 fibers and pores per group) showed that PHBV/PCL grafts with and without VEGF consisted mostly of nanoscale fibers/small pores and microscale fibers/large pores, respectively. Moreover, it revealed a lower mean fiber diameter and mean pore area in PHBV/PCL grafts with VEGF compared to those without. Data are represented as mean with standard deviation, ^∗∗∗^*P* < 0.001, two-tailed Student’s *t*-test.

Both PHBV/PCL and PHBV/PCL/VEGF grafts were highly porous (**Figures 1A,B**). However, PHBV/PCL grafts consisted mostly of microscale fibers and large pores while PHBV/PCL/VEGF grafts mainly constituted of nanoscale fibers and small pores (**Figures 1A,B**). Quantitative image analysis showed that PHBV/PCL/VEGF grafts had significantly lower mean fiber diameter and mean pore area compared to their unmodified counterparts (**Figure 1B**).

### Incorporation of VEGF Significantly Improves Physico-Mechanical Properties of PHBV/PCL Vascular Grafts 6 Months Postimplantation

**FIGURE 2:**
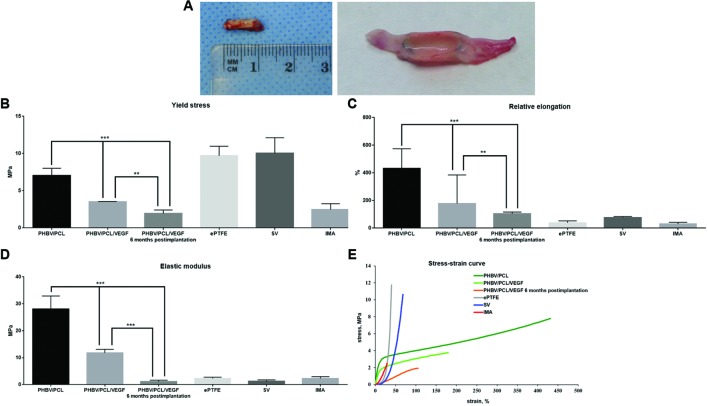
**Physico-mechanical properties of PHBV/PCL vascular grafts with and without VEGF. (A)** Macroscopic images of PHBV/PCL grafts with VEGF 6 months postimplantation; **(B)** Incorporation of VEGF decreased durability of PHBV/PCL grafts almost to the values of internal mammary artery, particularly 6 months postimplantation; **(C)** Incorporation of VEGF reduced elasticity of PHBV/PCL grafts, particularly 6 months postimplantation; **(D)** Incorporation of VEGF lowered stiffness of PHBV/PCL grafts, and 6 months postimplantation it was almost similar to the values of internal mammary artery; **(E)** Stress-strain curve of PHBV/PCL grafts with VEGF was more similar to that of internal mammary artery, particularly 6 months postimplantation. Data are represented as median with interquartile range, ^∗∗^*P* < 0.01, ^∗∗∗^*P* < 0.001, Mann–Whitney *U*-test.

In attempts to improve physico-mechanical properties of the grafts, we suggested that tissues that gradually replace degrading polymer may provide parameters similar to the native arteries. Therefore, we also tested PHBV/PCL/VEGF grafts 6 months postimplantation (**Figure 2A**) in addition to those before the implantation. We revealed that durability, elasticity, and stiffness of PHBV/PCL/VEGF grafts before the implantation were respectively 2-, 2.41-, and 2.37-fold lower compared to unmodified grafts but these values were still far from those of IMA (**Figures 2B–D**). However, PHBV/PCL/VEGF grafts explanted from rat abdominal aorta 6 months postimplantation demonstrated durability and stiffness almost similar to IMA; in addition, their elasticity and stress-strain curve were also closer to IMA than before the implantation (**Figures 2B–E**).

### Incorporation of VEGF Enhances Endothelialization, Improves Patency, and Recruits Cells to PHBV/PCL Vascular Grafts

**FIGURE 3:**
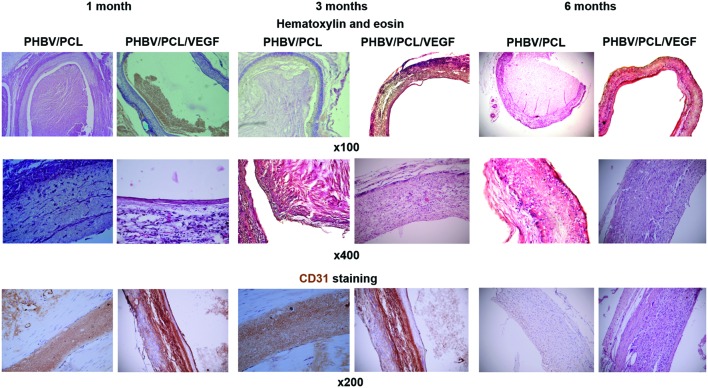
***In vivo* performance of PHBV/PCL vascular grafts with and without VEGF: histological and immunohistochemical examination**. PHBV/PCL grafts with VEGF were completely patent whereas those without were occluded. Hematoxylin and eosin staining revealed a putative endothelial cell monolayer in certain parts of the inner surface of PHBV/PCL grafts with VEGF even 1 month postimplantation and almost entire inner surface 3 and 6 months postimplantation. This was confirmed by CD31 staining but was not the case for PHBV/PCL grafts without VEGF. CD31-positive cells are stained brown.

**FIGURE 4:**
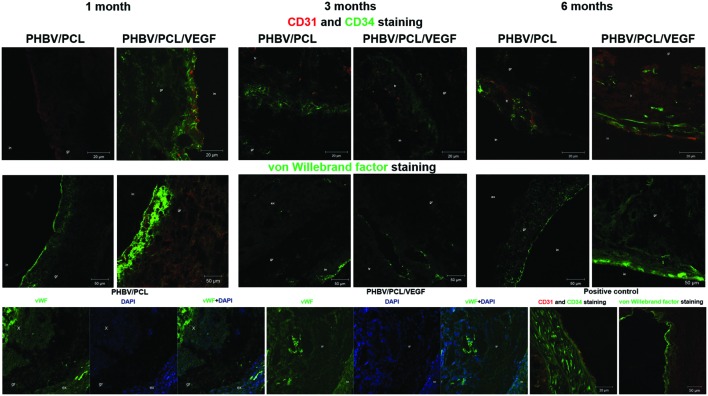
***In vivo* performance of PHBV/PCL vascular grafts with and without VEGF: confocal laser scanning microscopy**. PHBV/PCL grafts with VEGF contained many CD31-, CD34-, and vWF-positive cells which therefore were defined as endothelial cells but this was not the case for unmodified grafts. Furthermore, combined vWF- and DAPI staining revealed a significant increase in a total number of cells within the graft in PHBV/PCL/VEGF compared to PHBV/PCL grafts 6 months postimplantation. Native rat abdominal aorta was used as a positive control. CD31-positive cells are stained red while CD34 and vWF-positive cells are stained green. X, tr, in, gr, and ex are for neointima, thrombus, inner, middle, and upper third of the graft.

**FIGURE 5:**
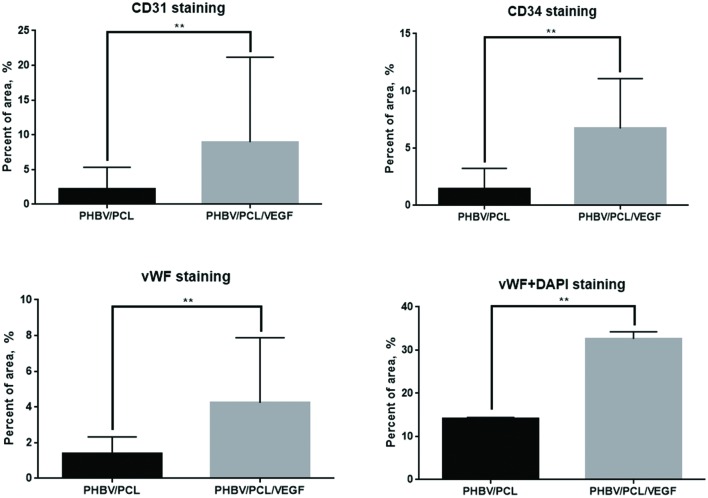
***In vivo* performance of PHBV/PCL vascular grafts with and without VEGF: quantitative image analysis**. Quantitative image analysis (three images per staining) confirmed an increase in number of CD31-, CD34, and vWF-positive cells in PHBV/PCL grafts with VEGF compared to those without after the implantation into rat abdominal aorta. In addition, this approach verified the increase in a total number of cells within the graft in PHBV/PCL/VEGF compared to PHBV/PCL grafts 6 months postimplantation. Data are represented as mean with standard deviation, ^∗∗^*P* < 0.01, two-tailed Student’s *t*-test.

With the aim to compare endothelialization rate of PHBV/PCL and PHBV/PCL/VEGF grafts, we stained grafts with (1) hematoxylin and eosin; (2) antibodies to CD31, CD34, and vWF which were previously defined as endothelial cell markers (Hristov et al., 2003). One month postimplantation, histological and immunohistochemical examination revealed a thrombus occluding the graft lumen or intimal hyperplasia but no or a few CD31-positive cells in all PHBV/PCL grafts (**Figure 3**). However, 3/4 (75%) PHBV/PCL/VEGF grafts were patent and had many CD31-positive cells at the inner surface (**Figure 3**). In addition, we identified macrophages, fibroblasts, and collagen fibers only in the outer third of PHBV/PCL grafts but in the entire graft wall of PHBV/PCL/VEGF grafts. Confocal laser scanning microscopy demonstrated that inner surface of all PHBV/PCL grafts contained only a few CD31-, CD34, and vWF-positive cells (**Figure 4**). However, we found many CD31-, CD34-, and vWF-positive cells in 3/4 (75%) of PHBV/PCL/VEGF grafts (**Figure 4**). Similar results were obtained 3 and 6 months postimplantation. Moreover, combined vWF and DAPI staining showed a significant increase in the total number of cells within PHBV/PCL/VEGF compared to PHBV/PCL grafts 6 months postimplantation. Quantitative image analysis confirmed the findings from confocal laser scanning microscopy examination (**Figure 5**).

## Discussion

High porosity of the vascular graft, nanoscale fiber diameter and small pore area promote cell migration to the graft and further formation of the endothelial cell monolayer after the implantation (Catto et al., 2014). These features of the polymer scaffold make it similar to extracellular matrix (Catto et al., 2014). Thin polymer fibers increase the area for the cell-scaffold interactions that, in turn, enhances cell adhesion and further cell metabolism (Sill and von Recum, 2008). This is of crucial significance since cell infiltration of the graft wall improves integration of the scaffold with the host tissue (Sachlos and Czernuszka, 2003).

PHBV/PCL grafts with VEGF had a highly porous structure and, in contrast to those without, consisted mostly of nanoscale fibers and small pores. This can be explained by the presence of water phase. Therefore, the composition of PHBV/PCL/VEGF grafts was more similar to extracellular matrix compared to unmodified grafts.

Physico-mechanical properties of PHBV/PCL grafts significantly differed from those of native blood vessels; however, incorporation of VEGF made them more similar to those of IMA, and it was particularly significant 6 months postimplantation. Thus, we suggest the absence of collagen, elastin, and glycosaminoglycans as the cause of differences in physico-mechanical properties between the polymer grafts before implantation and IMA. This also corresponds to the literature (L’Heureux et al., 2007).

Thrombosis and thromboembolism even despite anticoagulant therapy are the main problems associated with small-diameter synthetic vascular grafts (Tatterton et al., 2012). The reasons for this are low blood flow in small-diameter vessels and low thromboresistance of poly(ethylene terephthalate) and ePTFE, which are currently used for the preparation of synthetic vascular grafts (Tatterton et al., 2012). Formation of the endothelial monolayer at the inner surface of the vascular grafts may improve their long-term patency (L’Heureux et al., 1998). Previously, we detected an endothelial cell monolayer in only one-fourth of PHBV/PCL vascular grafts 1 year postimplantation using rat abdominal aorta replacement model as here (Antonova et al., 2015a). In this study, all unmodified grafts were occluded or had the signs of neointima formation. In contrast, three-fourths of PHBV/PCL/VEGF vascular grafts were completely patent and had an endothelial cell monolayer even 6 months postimplantation. Moreover, incorporation of VEGF enhanced migration of CD34-positive cells to the graft. This is of particular importance since CD34 is a marker of endothelial progenitor cells, and migration of CD34-positive cells to the vascular graft was previously observed both *in vitro* (Boyer et al., 2000) and *in vivo* (De Visscher et al., 2012). In addition, we found that incorporation of VEGF led to an increase of the total number of cells within the graft. Therefore, we suggest that VEGF promotes endothelialization and improves patency of the vascular graft *in vivo*.

Our data demonstrate that incorporation of VEGF improves biofunctionalization of PHBV/PCL vascular grafts. It makes graft more similar to extracellular matrix and brings its physico-mechanical properties closer to those of IMA. Furthermore, it stimulates migration of cells to the graft, promotes formation of endothelial cell monolayer *in situ*, and improves graft patency.

## Author Contributions

LA, AK, YK, OB, and LB conceived and designed the study; VS, EK, and ES fabricated the grafts; LA, VS, and TG performed morphological assessment; LA, AM, VS, and TG performed evaluation of physico-mechanical properties; AM, AS, EV, EK, and EA performed *in vivo* implantation; LA, VS, EK, AB, and GV performed histological examination and immunohistochemistry; LA, AK, and VS performed data analysis and wrote the manuscript.

## Conflict of Interest Statement

The authors declare that the research was conducted in the absence of any commercial or financial relationships that could be construed as a potential conflict of interest.
